# One-year functional outcomes of patients aged 80 years or more undergoing colonic cancer surgery: prospective, multicentre observational study

**DOI:** 10.1093/bjsopen/zrac094

**Published:** 2022-08-16

**Authors:** Susanna Niemeläinen, Heini Huhtala, Esa Jämsen, Jyrki Kössi, Jan Andersen, Anu Ehrlich, Eija Haukijärvi, Suvi Koikkalainen, Selja Koskensalo, Anne Mattila, Tarja Pinta, Mirjami Uotila-Nieminen, Hanna Vihervaara, Marja Hyöty

**Affiliations:** Department of Gastroenterology and Alimentary Tract Surgery, Tampere University Hospital, Tampere, Finland; Faculty of Social Sciences, Tampere University, Tampere, Finland; Faculty of Medicine, Helsinki University, Helsinki, Finland; Department of Surgery, Gerontology Research Center (GEREC), Tampere, Finland; Department of Surgery, Päijät-Häme Central Hospital, Lahti, Finland; Department of Surgery, Vaasa Central Hospital, Vaasa, Finland; Department of Abdominal Surgery, Helsinki University Hospital, Finland; Department of Gastroenterology and Alimentary Tract Surgery, Tampere University Hospital, Tampere, Finland; Department of Surgery, Satakunta Central Hospital, Pori, Finland; Faculty of Medicine, Helsinki University, Helsinki, Finland; Department of Abdominal Surgery, Helsinki University Hospital, Finland; Department of Surgery, Central Hospital of Central Finland, Jyväskylä, Finland; Department of Surgery, Seinäjoki Central Hospital, Seinäjoki, Finland; Department of Surgery, North Karelia Central Hospital, Joensuu, Finland; Division of Digestive Surgery and Urology, Turku University Hospital, Turku, Finland; Faculty of Medicine, Turku University, Turku, Finland; Department of Gastroenterology and Alimentary Tract Surgery, Tampere University Hospital, Tampere, Finland

## Abstract

**Background:**

Older patients are at high risk of experiencing delayed functional recovery after surgical treatment. This study aimed to identify factors that predict changes in the level of support for activities of daily living and mobility 1 year after colonic cancer surgery.

**Methods:**

This was a multicentre, observational study conforming to STROBE guidelines. The prospective data included pre-and postoperative mobility and need for support in daily activities, co-morbidities, onco-geriatric screening tool (G8), clinical frailty scale (CFS), operative data, and postoperative surgical outcomes.

**Results:**

A total of 167 patients aged 80 years or more with colonic cancer were recruited. After surgery, 30 per cent and 22 per cent of all patients had increased need for support and decreased motility. Multivariableanalysis with all patients demonstrated that preoperative support in daily activities outside the home (OR 3.23, 95 per cent c.i. 1.06 to 9.80, *P* = 0.039) was associated with an increased support at follow-up. A history of cognitive impairment (3.15, 1.06 to 9.34, *P* = 0.038) haemoglobin less than 120 g/l (7.48, 1.97 to 28.4, *P* = 0.003) and discharge to other medical facilities (4.72, 1.39 to 16.0, *P* = 0.013) were independently associated with declined mobility. With functionally independent patients, haemoglobin less than 120 g/l (8.31, 1.76 to 39.2, *P* = 0.008) and discharge to other medical facilities (4.38, 1.20 to 16.0, *P* = 0.026) were associated with declined mobility.

**Conclusion:**

Increased need for support before surgery, cognitive impairment, preoperative anaemia, and discharge to other medical facilities predicts an increased need for support or declined mobility 1 year after colonic cancer surgery. Preoperative assessment and optimization should focus on anaemia correction, nutritional status, and mobility with detailed rehabilitation plan.

## Teaser

Greater increased need for support before surgery, cognitive impairment, preoperative anaemia, and discharge to other medical facilities predicted an increased need for support or declined mobility 1 year after colonic cancer surgery. Preoperative assessment and optimization should especially focus on anaemia correction, nutritional status, and mobility with a detailed rehabilitation plan.

## Introduction

The number of individuals aged 80 years or older is expected to double worldwide from 2020 to 2040 due increasing life expectancy^1,2^. Individuals in this age group have more than a four-fold probability of having colonic cancer than those aged 60 years^3^. Radical surgery represents the most successful treatment option for localized colonic cancer^4^; however, older patients are at higher risk of experiencing postoperative complications and functional disability after surgical treatment^5,6^. Patients with co-morbidities, poor nutritional status, frailty, and declined functional/cognitive status are more vulnerable to adverse events, prolonged disability, and dependency^7,8^. The heterogeneity of patients in this age group can lead to different outcomes if managed surgically^9^; however, physically and cognitively fit patients have similar surgical and functional outcomes to younger cohorts^10,11^.

In a recent study of more than 2000 patients aged more than 80 years undergoing surgery (including 649 colorectal resections), 19–26 per cent experienced a functional decline in activities of daily living within 30 days after surgery; however, the respective proportion was markedly higher (40–47 per cent) in patients with preoperative malnutrition, postoperative delirium, or major complications^12^. Loss of independence, which included the need for new walking aids and increased care needs such as a discharge to other medical facilities, was reported in 67–84 per cent of patients aged 75 years or older after discharge from the hospital after the surgical operation^13^. Prolonged and even permanent decline in functional performance has been reported in 23–69 per cent of patients 1–2 years after colorectal surgery^14–16^.

Maintenance of physical function and functional independence is a highly valued determinant of quality of life for aged patients; therefore, such outcomes may be prioritized over surgical outcomes and cancer survival^17^. Most patients would choose less invasive treatment options to secure their functional independence^18^. Consequently, when choosing optimal, patient-centred cancer treatment for older patients, it is essential to understand the effect of surgery on postoperative functional performance and recovery in addition to cancer prognosis^19^.

Only one study has evaluated the risk of long-term functional disability of older patients after elective colonic cancer surgery. In a study of nursing home residents, Finlayson *et al*. reported that half of the surviving patients had a functional decline in activities of daily living 1 year after surgery. Residents aged 80 years or older were more than 50 per cent more likely than younger residents to experience a functional decline^16^.

This study aimed to identify factors that predict increased support in activities of daily living and decreased mobility 1 year after elective surgery in patients with stage I–III colonic cancer aged 80 years and older.

## Patients and methods

### Study design and population

A multicentre, prospective observational cohort study of patients aged 80 years or older with stage I–III colonic cancer was conducted in nine Finnish public hospitals. Recruitment started on 1 April 2019 and continued until 15 August 2020. Patients with metastatic disease, emergency operations, or life expectancy less than 6 months were excluded. In addition, patients who consented to the study but were treated non-operatively or found to have metastatic or benign disease at surgery were excluded from the present analysis. Our previously published articles have described the detailed study protocol, data collection, and short-term results^10,20^.

This study followed the STROBE guidelines^21^. The Ethics Committee of Tampere University Hospital (reference approval number R19028) and the institutional review boards at each study site approved the study protocol. The study was registered at http://www.clinicaltrials.gov (registration number NCT03904121) in April 2019.

### Outcomes

The primary outcome measures were the changes in support for activities of daily living and mobility 1 year after surgery. Performance in activities of daily living was categorized as being able to perform activities of daily living independently, with support outside the home, with support for housework or with support for basic activities of daily living. Mobility inside and outside the home was categorized as being able to move independently without an aid, moving independently with a walking aid, or being unable to move unassisted. Based on a comparison between preoperative and 1-year postoperative function, the performance of activities of daily living was classified as ‘same or with less support’ or ‘increased need for support’, and mobility as ‘same or better’ or ‘declined’. Patients who died during the follow-up time of 1 year were classified as those with an increased need for support or declined mobility.

### Statistical analysis

Demographic data and outcomes were expressed as percentages. The median and range were calculated for age, preoperative laboratory values, and BMI. The distribution of the predictive variables in case numbers and percentages or medians with interquartile ranges according to the outcome variables of support in daily activities and mobility 1 year after surgery respectively, were calculated. When appropriate, associations between the categorical variables were tested with the Pearson chi-square test or Fisher’s exact test for univariable analysis. Multivariable analyses of the factors associated with changes in performance with activities of daily living and mobility were carried out with binary logistic regression. Results are shown as odds ratios (OR) with a 95 per cent confidence interval (c.i.). All clinically (age, cognitive impairment, clinical frailty scale (CFS)^22^ and mini-nutritional assessment-short form (MNA-SF)^23^), and statistically significant variables (*P* < 0.05) in the univariate model were included in the multivariable model. Statistical analyses were performed with SPSS^®^ version 26 (IBM, Armonk, New York, USA). The analyses were performed for all patients and those patients who were independent before surgery in activities of daily living or able to move without assistance.

## Results

### Patients and clinical characteristics

Of the 250 eligible patients, 189 (76 per cent) patients consented to participate. Fourteen patients were treated non-operatively because of their age, withheld consent, had poor functional status, or the risks of anaesthesia. Eight patients were excluded because of metastatic or benign findings at surgery or in the pathological specimen. This left 167 patients for analysis in this study.

The median age was 84.5 (range 80–97) years, and 59 per cent were women. Before surgery, most patients lived at home (98 per cent), performed activities of daily living independently (54 per cent), were mobile without assistive devices (60 per cent), and moved unassisted outside the home (72 per cent). *Table 1* shows patients’ baseline characteristics.

**Table 1:** Baseline characteristics (*n* = 167)

	*n* (median)	% (range)
**Sex ratio (F:M)**	99/68	59.3/40.7
**Age, (years)**	84.5	80–97
**BMI, (kg/m^2^)**	25.7	16.5–40
**Living status**		
Home alone or with someone	164	98.2
Assisted living accommodation	3	1.8
**Need for support in activities of daily living**		
Independent	90	53.9
Outdoors independent	23	13.8
Out and indoors with housework	35	20.9
Out and indoors with basic activities	19	11.4
**Mobility**		
Independent	100	59.9
Independent with walking aid	60	35.9
Dependent of support care or unable to move	7	4.2
**Hospital admissions <6 months**		
One or more	80	48.5
**Co-morbidities**		
Hypertension	116	69.5
Cardiovascular disease	87	52.2
Diabetes	53	31.7
History of cognitive impairment	48	30.8
Renal insufficiency	31	18.6
Cerebrovascular disease	23	13.8
Pulmonary disease	18	10.8
**Polypharmacy (≥5 medications)**	101	60.5
**Charlson co-morbidity index**	6.0	4–15
**ASA score ≥3**	122	73.1
**G8 score**	12	5–16
**Clinical frailty scale**	3	1–8
**Mini-nutritional assessment-short form**	10	3–12
**Haemoglobin (g/l)**	112	66–169
**Albumin (g/l), (missing 14 values)**	34.0	21–50
**Estimated glomerular filtration rate**	61.7	19.8–93.1

Values are *n* (%).

Most of the operative procedures were performed for right-sided colonic cancer (62 per cent). An intended laparoscopic resection was performed in 112 patients (67 per cent), and 15 cases (13 per cent) were converted to open surgery due to anatomical or technical reasons. Overall, the postoperative complication rate was 40 per cent, and 13 per cent of all complications were severe. Surgical complications were reported for 23 per cent of patients. The most common surgical complications were ileus (13 per cent), anastomotic leakage (4.8 per cent), enterotomy (2.4 per cent), wound dehiscence (2.4 per cent), and superficial surgical site infections (1.8 per cent). The most common non-surgical complications were pulmonary (8 per cent) and cardiovascular (6 per cent). Both surgical and non-surgical complications occurred in 10 patients (6 per cent).

The median duration of hospital stay in the operating hospital was 5 days (range 2–36 days). Ninety-one patients (54 per cent) were discharged from operating hospitals to home and the rest to other medical facilities. Within 30 days of discharge, readmission occurred in 14 patients (8.4 per cent). The overall 30-day and 1-year mortality rates were 1.8 per cent (3 of 167) and 6.6 per cent (11 of 167) respectively. The main causes of death within 1 year were cardiopulmonary (36 per cent), colonic cancer (27 per cent), and surgery-related (18 per cent) reasons.

### Postoperative functional status

**Fig. 1:**
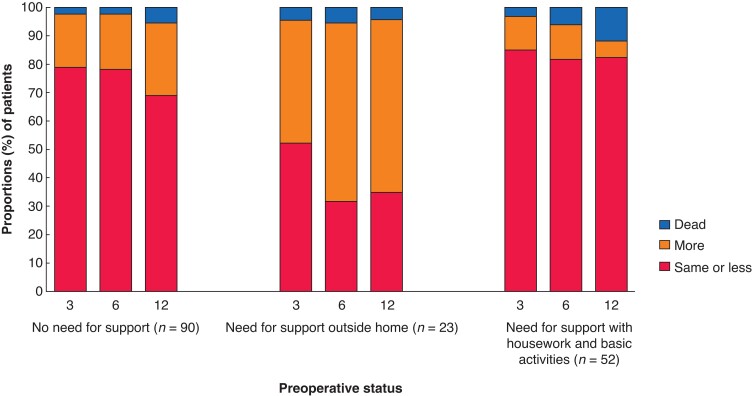
Changes in need for support with activities in daily living at 3, 6, and 12 months after curatively aimed colonic cancer surgery (stage I–III) for patients aged 80 years or more

After excluding the patients who had died or where data were not available, the proportion of patients living at home after surgery were 94 per cent (148 patients, 1 month), 97 per cent (147 patients, 3 months), 94 per cent (149 patients, 6 months), and 94 per cent (137 patients, 1 year). Increased support in activities of daily living was needed by 27 per cent (43 patients of 159, 1 month), 22 per cent (35 patients of 159, 3 months), 25 per cent (35 patients of 153, 6 months), and 30 per cent (50 patients of 165, 1 year). *Fig. 1* shows postoperative changes according to three different preoperative dependency levels. After 1 year, 31 per cent (28 of 90) of independent patients needed more support in activities of daily living. The 1-year mortality rate for those who, before surgery, performed activities of daily living independently, with support outside the home, with support for housework or support in basic activities of daily living, were 5.6 per cent, 4.3 per cent, 11.8 per cent and 5.6 per cent respectively (*P* = 0.635).

**Fig. 2:**
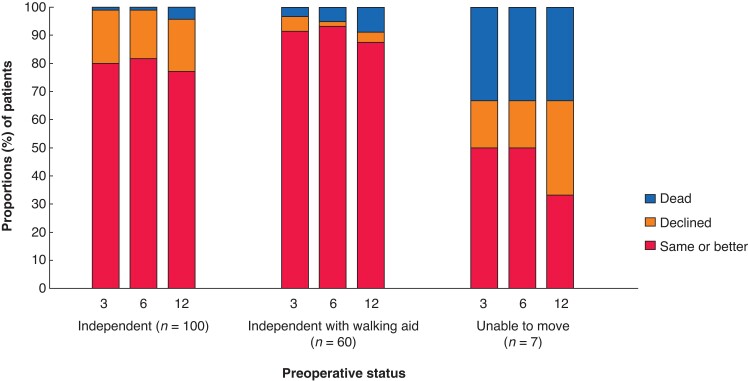
Changes in mobility at 3, 6, and 12 months after curatively aimed colonic cancer surgery (stage I–III) for patients aged 80 years or more

Reduced postoperative mobility was reported with 18 per cent (28 patients of 160, 1 month), 17 per cent (27 patients of 159, 3 months), 15 per cent (25 patients of 163, 6 months), and 22 per cent (35 patients of 157, 1 year). A new walking aid was needed by 16 per cent of patients 1 year after surgery. *Figure 2* shows postoperative changes in mobility according to different preoperative mobility levels. After 1 year, 25 per cent (24 of 95) of patients who were fully mobile before surgery had declined mobility. The 1-year mortality rates for mobile patients who were independently mobile before surgery with or without a walking aid or unable to move unassisted were 4.3 per cent, 8.9 per cent and 33 per cent respectively (*P* = 0.039).

### Factors associated with changes in support in activities of daily living 1 year after surgery

At 1-year follow-up, patients who required more support for activities of daily living had increased history of preoperative cognitive impairment (44 per cent *versus* 24 per cent, *P* = 0.010), were dependent on support outside the home (65 per cent *versus* 31 per cent, *P* < 0.001) but had diabetes less often (15 per cent *versus* 38 per cent, *P* = 0.006). G8 score less than 12 compared with G8 score more than 14 (35 per cent *versus* 8 per cent, *P* = 0.147), MNA-SF 0-7 compared with MNA-SF score of 14 or more (32 per cent *versus* 6 per cent, *P* = 0.087), and open surgery compared with laparoscopy (37 per cent *versus* 27 per cent, *P* = 0.101) had a trend towards increased need for support for activities of daily living, but were not statistically significant (*Table 2* and *Table S1*). Among patients who were independent before surgery, those who needed more support at follow-up more often were women (42 per cent *versus* 20 per cent, *P* = 0.023), had COPD (80 per cent *versus* 28 per cent, *P* = 0.031), asthma (80 per cent *versus* 28 per cent, *P* = 0.029), and open surgery (53 per cent *versus* 25 per cent, *P* = 0.029) but had diabetes less often (9 per cent *versus* 39 per cent, *P* = 0.006) (*Table S2*).

**Table 2:** Selected factors evaluating the need for postoperative support in activities of daily living 1 year after surgery

	All patients *n* = 165	Same or less *n* = 115	*P*
**Age, (years)**			0.802
80–84	92	65 (70.7)	
85–89	51	36 (70.6)	
≥90	22	14 (63.6)	
**Preoperative need for support in activities of daily living**			<0.001
No	90	62 (68.9)	
Yes, outside home	23	8 (34.8)	
Yes, with housework	34	28 (82.4)	
Yes, with basic activities	18	17 (94.4)	
**Preoperative mobility**			0.555
Independent	100	73 (73.0)	
Independent with walking aid	60	38 (63.3)	
Dependent	5	4 (80.0)	
**Preoperative cognitive impairment**			0.010
No	114	87 (76.3)	
Yes	50	28 (56.0)	
**Charlson co-morbidity index**			0.730
4–6	99	70 (70.7)	
>6	66	45 (68.2)	
**G8 score**			0.147
<12	75	49 (65.3)	
12–14	77	54 (70.1)	
>14	13	12 (92.3)	
**Clinical frailty scale**			0.644
1–2	38	25 (65.8)	
3–4	85	62 (72.9)	
5–9	42	28 (66.7)	
**Mini-nutritional assessment-short form**			0.087
0–7	38	26 (68.4)	
8–11	111	74 (66.7)	
≥12	16	15 (93.8)	
**Haemoglobin (g/l)**			0.263
≤120	105	70 (66.7)	
>120	60	45 (75.0)	
**Type of operation**			0.101
Laparoscopy	111	81 (73.0)	
Open	54	34 (63.0)	
Conversion	15	12 (80.0)	
**Surgical complications**			0.355
No	125	86 (67.7)	
Yes	37	28 (75.7)	
**Non-surgical complications**			0.721
No	126	89 (70.6)	
Yes	37	25 (67.6)	
**Discharge from operating hospital** *			0.154
Home	91	67 (73.6)	
Other medical facilities	73	48 (65.8)	

Values are *n* (%).

* Death during hospital stay (*n* = 1). Missing data (*n* = 2).

In multivariable logistic regression analysis, preoperative need for support in activities of daily living outside the home (*P* = 0.039) was independently associated with an increased need for support at follow-up. Conversely, diabetes was associated with a lower probability of increased care needs with all patients (*P* = 0.016) and patients who were independent before surgery (*P* = 0.026) (*Table 3* and *Table S3*).

**Table 3:** Factors influencing increased support in activities of daily living and declined mobility 1 year after surgery in multivariable analysis (binary logistic regression)

	OR	95% c.i.	*P*
**Support in activities of daily living**			
Age, (years)			
80–84	1		
85–89	0.83	0.34–1.98	0.668
≥90	1.38	0.43–4.49	0.590
Preoperative support in activities of daily living			
Independent	1		
Outside assistance	3.23	1.06–9.80	0.039
Dependent	0.19	0.06–0.62	0.006
History of cognitive impairment	1.90	0.82–4.39	0.133
Diabetes	0.30	0.11–0.80	0.016
Clinical frailty scale*	1.20	0.84–1.70	0.316
Mini-nutritional assessment-short form*	0.85	0.70–1.03	0.093
**Mobility**			
Age, (years)			
80–84	1		
85–89	0.69	0.21–2.28	0.547
≥90	0.50	0.11–2.29	0.369
Preoperative mobility			
Independent	1		
With walking aid	0.19	0.05–0.74	0.016
Dependent	0.89	0.03–24.0	0.945
History of cognitive impairment	3.31	1.09–10.1	0.035
Clinical frailty scale*	1.19	0.68–2.08	0.548
Mini-nutritional assessment-short form*	0.92	0.72–1.17	0.499
Anaemia (<120 g/l)	8.08	2.05–31.8	0.003
Open surgery (compared with laparoscopy)	2.52	0.81–7.88	0.111
Surgical complications	2.53	0.87–7.33	0.088
Non-surgical complications	1.91	0.57–6.37	0.295
Discharge to other medical facilities	3.64	1.07–12.4	0.038

* Continuous.

### Factors associated with changes in mobility 1 year after surgery

At 1 year after surgery, declined mobility was seen more often in patients who had preoperative haemoglobin levels less than 120 g/l (29 per cent *versus* 11 per cent, *P* = 0.013), open surgery (31 per cent *versus* 18 per cent, *P* = 0.010), surgical or non-surgical complications (34 per cent *versus* 19 per cent, *P* = 0.046 and 42 per cent *versus* 17 per cent, *P* = 0.002 respectively), or had been discharged to other medical facilities after surgery (34 per cent *versus* 12 per cent, *P* < 0.001). In addition, independently mobile patients more often had declined motility in comparison with mobile patients with a walking aid (26 per cent *versus* 14 per cent, *P* = 0.030) (*Table 4* and *Table S4*). Among patients who were fully mobile before surgery, those who experienced declined mobility, had preoperative haemoglobin levels less than 120 g/l (36 per cent *versus* 8 per cent, *P* = 0.003), MNA-SF 0-7 compared with MNA-SF scores of 14 or more (44 per cent *versus* 0 per cent, *P* = 0.025), open surgery (50 per cent *versus* 18 per cent, *P* = 0.004), and were discharged to other medical facilities (40 per cent *versus* 17 per cent, *P* = 0.012) (*Table S5*).

**Table 4:** Selected factors evaluating postoperative mobility 1 year after surgery

	All patients *n* = 157	Same or better *n* = 122	*P*
**Age, (years)**			0.872
80–84	87	68 (8.2)	
85–89	49	37 (75.5)	
≥90	21	17 (81.0)	
**Preoperative need for support in activities of daily living**			0.776
No	84	67 (79.8)	
Yes, outside home	22	17 (77.3)	
Yes, with housework	33	23 (69.7)	
Yes, with basic activities	18	15 (83.3)	
**Preoperative mobility**			0.030
Independently	95	71 (75.5)	
Independently with walking aid	57	49 (86.0)	
Dependent	5	2 (40.0)	
**Preoperative cognitive impairment**			0.137
No	108	88 (81.5)	
Yes	48	34 (70.8)	
**Charlson co-morbidity index**			0.859
4–6	94	74 (78.7)	
>6	63	48 (76.2)	
**G8 score**			0.055
<12	71	49 (69.0)	
12–14	76	65 (85.5)	
>14	10	8 (80.0)	
**Clinical frailty scale**			0.645
1–2	37	29 (78.4)	
3–4	80	64 (80.0)	
5–9	40	29 (72.5)	
**Mini-nutritional assessment-short form**			0.067
0–7	35	24 (68.6)	
8–11	109	85 (78.0)	
≥12	13	13 (100)	
**Haemoglobin (g/l)**			0.013
≤120	101	72 (71.3)	
>120	56	50 (89.3)	
**Type of operation**			0.010
Laparoscopy	106	87 (82.1)	
Open	51	35 (68.6)	
Conversion	13	12 (92.3)	
**Surgical complications**			0.046
No	122	99 (81.8)	
Yes	35	23 (65.7)	
**Non-surgical complications**			0.002
No	119	99 (83.2)	
Yes	36	21 (58.3)	
**Discharge from operating hospital** *			<0.001
Home	86	76 (88.4)	
Other medical facilities	70	46 (65.7)	

Values are *n* (%).

* Death during hospital stay (*n* = 1). Missing data (*n* = 10).

In multivariable logistic regression analysis, a history of cognitive impairment (*P* = 0.035), preoperative haemoglobin level less than 120 g/l (*P* = 0.003), and discharge after surgery to other medical facilities (*P* = 0.038) were independently associated with declined mobility. Conversely, being mobile before surgery with a walking aid was associated with a diminished risk of declined mobility (*P* = 0.016) (*Table 3*). In patients who were fully mobile before surgery, preoperative haemoglobin level less than 120 g/l (*P* = 0.008) and discharge to other medical facilities (*P* = 0.026) were significantly associated with declined mobility (*Table S3*).

## Discussion

This national multicentre study analysed the impact of colonic cancer surgery on functionally independent patients aged 80 years or more than 1 year after the elective operation. This study suggests that 69 per cent of patients who were independent in daily activities before surgery maintained their independence well over the first year. Similarly, 76 per cent of patients who were fully mobile before surgery maintained their mobility. Decline in functional performance and mobility were associated with existing impairments, indicating that physical and cognitively independent fit patients achieve satisfactory recovery in 1 year of follow-up; however, comprehensive preoperative evaluation and optimization may be advisable even for the fittest patients to identify anaemia and malnutrition, both of which were also associated with poorer outcomes.

The systemic stress reaction associated with major abdominal surgery causes a reduction in older individuals’ physiological and functional capacity. Lawrence *et al*. reported that in patients older than 65 years undergoing general surgery, postoperative recovery with activities of daily living and with housework took up to 3 and 6 months respectively. Prolonged recovery was more often seen with patients with declined preoperative physical performance status and severe complications^24^. The postoperative reduction in physiological reserve is caused by inflammation and tissue damage with changes in metabolic function^25^. With age-related physiological changes in major organs, the impact of surgery is thus more damaging in older patients^5^, especially for those with preoperative declined functional or cognitive status, frailty, and postoperative complications^26,27^.

This study showed a 40 per cent postoperative complication rate, and 13 per cent of all complications were severe. Open surgery and postoperative complications were associated with a reduction in long-term mobility. In previous studies, severe postoperative complications have also been associated with mortality^28,29^; however, the 6.6 per cent 1-year mortality rate for all patients was low, showing convergent development with earlier studies^10,30^. The enhanced recovery after surgery programme and minimally invasive surgery, which have been shown to benefit postoperative outcomes, were well established at the recruiting hospitals^31^. Consequently, comprehensive preoperative assessment of risks for adverse events and allowing sufficient time for patients’ preoperative medical optimization is recommended. In addition, intensive mobility rehabilitation should be initiated as soon as possible.

Of the study population, 55 per cent of patients were independent before surgery without any outside support with activities of daily living, and 69 per cent of them maintained independence 1 year after surgery; however, fit women and patients needing mobility assistance outdoors might benefit from further evaluation to exclude geriatric syndromes. On the contrary, 65 per cent of patients dependent on external support outside the home had increased demand for support with activities of daily living 1 year after surgery. Those patients may already have had other co-morbidities and frailty that, before surgery, impaired functional performance. In this group, major cancer surgery might have worsened the surgical stress reaction causing significant postoperative functional decline, protracted recovery, and possible permanent disability. These findings suggest that patients dependent on support in activities of daily living might benefit from preoperative risk evaluation and structured geriatric assessment to recognize conditions such as frailty, malnutrition, and cognitive impairment^8,19^.

At 1 year, mobility had declined by 24 per cent in mobile-independent patients and by 14 per cent in patients with walking aids compared with 60 per cent in mobile-dependent patients. In addition, immobile patients had an eight-times greater risk of 1-year mortality compared with mobile patients (4 per cent *versus* 33 per cent). Preoperative mobility with functionally independent patients with or without walking aid was slightly decreased during the first 3 months after treatment but flattened out after that, showing recovery during the first year after major surgery. These findings emphasize that maintaining mobility even with the aid of a new mobility device is essential for satisfactory long-term mobility recovery from cancer surgery; however, a greater proportion of patients discharged to other medical facilities had a loss of independence and impaired mobility. This might be a long-term consequence from patient’s preoperative status, but it may also indicate incomplete early mobilization and rehabilitation after surgery. In addition, immobilization increases the risk of sarcopenia, leading to further delay in rehabilitation^32^. To avoid adverse effects of these factors on long-term mobility, a personalized rehabilitation programme designed before surgery, including a discharge plan might be beneficial.

Patients with preoperative haemoglobin level less than 120 g/l had reduced mobility, suggesting that careful preoperative anaemia assessment and correction is essential before surgery for preservation and enhancement of postoperative mobility^33^. In addition, those with impaired nutritional status, according to MNA-SF, low BMI, and albumin values, seemed to be associated with a declined mobility, advocating nutritional supplements to optimize the nutritional status before and after surgery^34^.

Patients with impaired cognitive function and memory loss before surgery had poorer mobility and increased support for activities in daily living after surgery, in keeping with a recent study^35^. In the present study, the preoperative data showed a discrepancy between self-reported memory decline and diagnosed dementia reported earlier with the same cohort^10^. These findings emphasize that preoperative cognitive disturbances are more frequent with older individuals, and awareness of possible impairment requires cautious preoperative assessment. Consequently, the use of easily adopted cognitive tests, such as the mini-mental state examination, are recommended before surgery^36^.

Comprehensive geriatric assessment (CGA) evaluates patients’ co-morbidities, polypharmacy, physical performance, cognitive impairment, functional, nutritional and emotional status, and social support^11^. CGA may be beneficial in identifying patients who are at risk of an adverse event; however, in surgical units where current resource limitations make CGA challenging, rapid screening tools for frailty, nutritional and functional prehabilitation may be advantageous^10,37^. During the study interval, some hospitals had established perioperative collaborations with geriatricians, who are increasingly participating as members at colorectal multidisciplinary teams before the commencement of cancer treatment.

The strengths of this study included a prospectively collected nationwide representative cohort. As Finland follows uniform and standardized protocols for colonic cancer surgery, the data population was homogenous and comprehensive^38^. The study equates to standard everyday surgical practice, so the outcomes are relevant with less selection bias and more relevance to real-life settings. There are some limitations to this study. The present study results were based on patient-reported questionnaires regarding the preoperative need for support with activities of daily living, mobility, and cognitive impairment, and no validated geriatric evaluation was conducted. Lack of more-detailed functional measure scales, such as the six-item activities of daily living and eight-item instrumental activities of daily living index, precluded reporting more-detailed functional changes. Almost half the patients were discharged from the surgical ward to other medical facilities instead of home, so the total duration of hospital stay and thus an excess need for supportive care and mobility decline with loss of independence was unknown. Quality of life before and after surgery was not measured.

Increased need for support in daily living before surgery, cognitive impairment, preoperative anaemia, and discharge after surgery to other medical facilities predict an increased need for support or declined mobility 1 year after colonic cancer surgery. Preoperative assessment and optimization should focus on anaemia correction, nutritional status, and mobility with a detailed rehabilitation plan.

## Supplementary Material

zrac094_Supplementary_Data

## Data Availability

The data sets generated or analysed during the present study are not publicly available due to Finnish laws privacy protection.
