# Supplementary material for: One-year functional outcomes of patients aged 80 years or more undergoing colonic cancer surgery: prospective, multicentre observational study
Source: BJS Open. 2022 Aug 16;6(4):zrac094. doi: 10.1093/bjsopen/zrac094 (PMC9380996; doi:10.1093/bjsopen/zrac094)
Supplement: zrac094_Supplementary_Data [file zrac094_supplementary_data.docx]

| **Table S1** Factors, which were evaluated for the need for postoperative support in activities of daily living one-year after surgery. Missing data (n=2). | | | |
| --- | --- | --- | --- |
|  | **All patients n=165** | **Same or less**  **n=115** | **p-value** |
|  |  | **n (%)** |  |
| Age, years |  |  | 0.802 |
| 80-84 | 92 | 65 (70.7) |  |
| 85-89 | 51 | 36 (70.6) |  |
| 90- | 22 | 14 (63.6) |  |
| Gender |  |  | 0.138 |
| Female | 98 | 64 (65.3) |  |
| Male | 67 | 51 (76.1) |  |
| BMI, kg/m^2^ |  |  | 0.191 |
| < 24 | 59 | 36 (61.0) |  |
| 24-29 | 69 | 51 (73.9) |  |
| > 29 | 37 | 28 (75.7) |  |
| Living status |  |  | 0.554 |
| Home | 162 | 112 (69.1) |  |
| Assisted living accommodation | 3 | 3 (100.0) |  |
| Hospital admissions < 6 months |  |  | 0.199 |
| No | 84 | 63 (75.0) |  |
| Yes | 79 | 52 (65.8) |  |
| Preoperative need for support in activities of daily living |  |  | <0.001 |
| No | 90 | 62 (68.9) |  |
| Yes, outside home | 23 | 8 (34.8) |  |
| Yes, with housework | 34 | 28 (82.4) |  |
| Yes, with basic activities | 18 | 17 (94.4) |  |
| Preoperative mobility |  |  | 0.555 |
| Independent | 100 | 73 (73.0) |  |
| Independent with walking aid | 60 | 38 (63.3) |  |
| Dependent | 5 | 4 (80.0) |  |
| Preoperative mobility outdoors |  |  | 0.337 |
| Yes | 121 | 85 (70.2) |  |
| Assisted | 40 | 26 (65.0) |  |
| No | 4 | 4 (100.0) |  |
| Use of walking aid |  |  | 0.299 |
| No | 86 | 63 (73.3) |  |
| Yes | 79 | 52 (65.8) |  |
| Diagnosis of |  |  |  |
| Congestive heart disease |  |  | 0.815 |
| No | 126 | 88 (69.8) |  |
| Yes | 30 | 20 (66.7) |  |
| Coronary disease |  |  | 0.459 |
| No | 122 | 86 (70.5) |  |
| Yes | 35 | 25 (71.4) |  |
| Hypertension |  |  | 0.223 |
| No | 46 | 29 (63.0) |  |
| Yes | 114 | 83 (72.8) |  |
| Chronic obstructive pulmonary disease (COPD) |  |  | 0.200 |
| No | 155 | 110 (71.0) |  |
| Yes | 7 | 3 (42.9) |  |
| Asthma |  |  | 0.072 |
| No | 151 | 109 (72.2) |  |
| Yes | 11 | 5 (45.5) |  |
| Diabetes |  |  | 0.006 |
| No | 112 | 70 (62.5) |  |
| Yes | 52 | 44 (84.6) |  |
| Cerebral stroke |  |  | 0.962 |
| No | 139 | 96 (69.1) |  |
| Yes | 23 | 16 (69.6) |  |
| Preoperative cognitive impairment |  |  | 0.010 |
| No | 114 | 87 (76.3) |  |
| Yes | 50 | 28 (56.0) |  |
| Number of medications |  |  | 1.000 |
| 0-4 | 66 | 46 (69.7) |  |
| ≥5 | 99 | 69 (69.7) |  |
| G8 score |  |  | 0.147 |
| < 12 | 75 | 49 (65.3) |  |
| 12-14 | 77 | 54 (70.1) |  |
| >14 | 13 | 12 (92.3) |  |
| Clinical Frailty Scale (CFS) |  |  | 0.644 |
| 1-2 | 38 | 25 (65.8) |  |
| 3-4 | 85 | 62 (72.9) |  |
| 5-9 | 42 | 28 (66.7) |  |
| Charlson Comorbidity Index (CCI) |  |  | 0.730 |
| 4-6 | 99 | 70 (70.7) |  |
| >6 | 66 | 45 (68.2) |  |
| ASA score |  |  | 0.492 |
| 2 | 43 | 27 (62.8) |  |
| 3 | 109 | 79 (72.5) |  |
| 4 | 11 | 8 (72.7) |  |
| Mini-Nutritional Assessment-Short Form |  |  | 0.087 |
| 0-7 | 38 | 26 (68.4) |  |
| 8-11 | 111 | 74 (66.7) |  |
| ≥ 12 | 16 | 15 (93.8) |  |
| Haemoglobin (g/L) |  |  | 0.263 |
| ≤ 120 | 105 | 70 (66.7) |  |
| > 120 | 60 | 45 (75.0) |  |
| Albumin (g/L) |  |  | 0.630 |
| ≤ 30 | 29 | 19 (65.5) |  |
| 31-34 | 49 | 36 (73.5) |  |
| ≥ 35 | 73 | 48 (65.8) |  |
| Estimated glomerular filtration rate (GFR) (ml/min) |  |  | 0.923 |
| < 45 | 37 | 25 (67.6) |  |
| 45-60 | 39 | 28 (71.8) |  |
| > 60 | 89 | 62 (69.7) |  |
| Type of operation |  |  | 0.101 |
| Laparoscopy | 111 | 81 (73.0) |  |
| Open | 54 | 34 (63.0) |  |
| Conversion | 15 | 12 (80.0) |  |
| Surgical complications |  |  | 0.355 |
| No | 125 | 86 (67.7) |  |
| Yes | 37 | 28 (75.7) |  |
| Non-surgical complications |  |  | 0.721 |
| No | 126 | 89 (70.6) |  |
| Yes | 37 | 25 (67.6) |  |
| Severe complication (CD III-IV) |  |  | 0.975 |
| No | 145 | 101 (69.7) |  |
| Yes | 20 | 14 (70.0) |  |
| Discharge from operating hospital^*^ |  |  | 0.154 |
| Home | 91 | 67 (73.6) |  |
| Other medical facilities | 73 | 48 (65.8) |  |
| Readmission |  |  | 0.865 |
| No | 143 | 99 |  |
| Yes | 14 | 10 |  |

*Death during hospital stay (n=1)

| **Table S2** Factors, which were evaluated for the need for postoperative support in activities of daily living with independent patients one-year after surgery. | | | |
| --- | --- | --- | --- |
|  | **All patients n=90** | **Same or less n=62** | **p-value** |
|  |  | **n (%)** |  |
| Age, years |  |  | 0.922 |
| 80-84 | 56 | 38 (67.9) |  |
| 85-89 | 25 | 18 (72.0) |  |
| 90- | 9 | 6 (66.7) |  |
| Gender |  |  | 0.023 |
| Female | 45 | 26 (57.8) |  |
| Male | 45 | 36 (80.0) |  |
| BMI, kg/m^2^ |  |  | 0.524 |
| < 24 | 35 | 22 (62.9) |  |
| 24-29 | 37 | 26 (70.3) |  |
| > 29 | 18 | 14 (77.8) |  |
| Hospital admissions < 6 months |  |  | 0.428 |
| No | 53 | 39 (73.6) |  |
| Yes | 35 | 23 (65.7) |  |
| Diagnosis of |  |  |  |
| Congestive heart disease |  |  | 0.437 |
| No | 76 | 52 (68.4) |  |
| Yes | 9 | 5 (55.6) |  |
| Coronary disease |  |  | 0.132 |
| No | 72 | 47 (65.3) |  |
| Yes | 14 | 12 (85.7) |  |
| Hypertension |  |  | 0.213 |
| No | 28 | 17 (60.7) |  |
| Yes | 61 | 45 (73.8) |  |
| Chronic obstructive pulmonary disease (COPD) |  |  | 0.031 |
| No | 82 | 59 (72.0) |  |
| Yes | 5 | 1 (20.0) |  |
| Asthma |  |  | 0.029 |
| No | 83 | 60 (72.3) |  |
| Yes | 5 | 1 (20.0) |  |
| Diabetes |  |  | 0.006 |
| No | 66 | 40 (60.6) |  |
| Yes | 23 | 21 (91.3) |  |
| Cerebral stroke |  |  | 0.171 |
| No | 83 | 55 (66.3) |  |
| Yes | 6 | 6 (100.0) |  |
| Preoperative cognitive impairment |  |  | 0.430 |
| No | 69 | 49 (71.0) |  |
| Yes | 21 | 13 (61.9) |  |
| Number of medications |  |  | 0.887 |
| 0-4 | 46 | 32 (69.6) |  |
| ≥5 | 44 | 30 (68.2) |  |
| G8 score |  |  | 0.222 |
| < 12 | 24 | 15 (62.5) |  |
| 12-14 | 55 | 37 (67.3) |  |
| >14 | 11 | 10 (90.9) |  |
| Clinical Frailty Scale (CFS) |  |  | 0.156 |
| 1-2 | 30 | 19 (63.3) |  |
| 3-4 | 55 | 41 (74.5) |  |
| 5-9 | 5 | 2 (40.0) |  |
| Charlson Comorbidity Index (CCI) |  |  | 0.766 |
| 4-6 | 63 | 44 (69.8) |  |
| >6 | 27 | 18 (66.7) |  |
| ASA score |  |  | 0.739 |
| 2 | 31 | 19 (61.3) |  |
| 3 | 54 | 39 (72.2) |  |
| 4 | 4 | 3 (75.0) |  |
| Mini-Nutritional Assessment-Short Form |  |  | 0.082 |
| 0-7 | 15 | 10 (66.7) |  |
| 8-11 | 60 | 38 (63.3) |  |
| ≥ 12 | 15 | 14 (93.3) |  |
| Haemoglobin (g/L) |  |  | 0.263 |
| ≤ 120 | 50 | 32 (64.0) |  |
| > 120 | 40 | 30 (75.0) |  |
| Albumin (g/L) |  |  | 0.892 |
| ≤ 30 | 11 | 7 (63.6) |  |
| 31-34 | 26 | 17 (65.4) |  |
| ≥ 35 | 43 | 30 (69.8) |  |
| Estimated glomerular filtration rate (GFR) (ml/min) |  |  | 0.247 |
| < 45 | 19 | 16 (84.2) |  |
| 45-60 | 19 | 13 (68.4) |  |
| > 60 | 52 | 33 (63.5) |  |
| Type of operation |  |  | 0.029 |
| Laparoscopy / conversion | 71 | 53 (74.6) |  |
| Open | 19 | 9 (47.4) |  |
| Surgical complications |  |  | 0.960 |
| No | 71 | 49 (69.0) |  |
| Yes | 19 | 13 (68.4) |  |
| Non-surgical complications |  |  | 0.748 |
| No | 76 | 52 (68.4) |  |
| Yes | 12 | 9 (75.0) |  |
| Severe complication (CD III-IV) |  |  | 0.495 |
| No | 80 | 56 (70.0) |  |
| Yes | 10 | 6 (60.0) |  |
| Discharge from hospital |  |  | 0.421 |
| Home | 60 | 43 (71.7) |  |
| Other medical facilities | 30 | 19 (63.3) |  |
| Readmission |  |  | 1.000 |
| No | 79 | 54 (68.4) |  |
| Yes | 8 | 6 (75.0) |  |

**Table S3** Factors influencing increased support in activities of daily living and declined mobility with independent patients one year after surgery in multivariable analysis (binary logistic regression).

|  | **OR** | | **95% CI** | **p-value** |
| --- | --- | --- | --- | --- |
| **Support in activities of daily living** |  | |  |  |
| Female gender | | 2.55 | 0.88-7.41 | 0.086 |
| Age | |  |  |  |
| 80-84 | | 1 |  |  |
| 85-89 | | 1.50 | 0.28-8.01 | 0.638 |
| ≥90 | | 0.96 | 0.15-6.02 | 0.963 |
| History of cognitive impairment | | 1.10 | 0.34-3.55 | 0.871 |
| Diabetes | | 0.15 | 0.03-0.80 | 0.026 |
| Clinical Frailty Scale (CFS)^*^ | | 1.24 | 0.71-2.19 | 0.452 |
| Mini Nutritional Assessment – Short Form^*^ | | 0.84 | 0.65-1.09 | 0.185 |
| Open surgery (compared to laparoscopy) | | 1.98 | 0.60-6.51 | 0.261 |
| **Mobility** | |  |  |  |
| Age | |  |  |  |
| 80-84 | | 1 |  |  |
| 85-89 | | 11.3 | 0.83-154.4 | 0.069 |
| ≥90 | | 6.94 | 0.49-99.1 | 0.153 |
| History of cognitive impairment | | 2.31 | 0.63-8.45 | 0.208 |
| Clinical Frailty Scale (CFS)* | | 0.92 | 0.48-1.76 | 0.798 |
| Mini Nutritional Assessment – Short Form* | | 0.88 | 0.66-1.17 | 0.374 |
| Anaemia (<120 g/l) | | 8.31 | 1.76-39.2 | 0.008 |
| Open surgery (compared to laparoscopy) | | 3.64 | 0.93-14.2 | 0.063 |
| Discharge to other medical facilities | | 4.38 | 1.20-16.0 | 0.026 |

*Continuous

| **Table S4** Factors which were evaluated for postoperative mobility one-year after surgery. Missing data (n=10) | | | |  |  |  |
| --- | --- | --- | --- | --- | --- | --- |
|  | **All patients n=157** | **Same or better n=122** | **p-value** |  |  |  |
|  |  | **n (%)** |  |  |  |  |
| Age, years |  |  | 0.872 |  |  |  |
| 80-84 | 87 | 68 (78.2) |  |  |  |  |
| 85-89 | 49 | 37 (75.5) |  |  |  |  |
| 90- | 21 | 17 (81.0) |  |  |  |  |
| Gender |  |  | 0.747 |  |  |  |
| Female | 95 | 73 (76.8) |  |  |  |  |
| Male | 62 | 49 (79.0) |  |  |  |  |
| BMI, kg/m^2^ |  |  | 0.311 |  |  |  |
| < 24 | 57 | 41 (71.9) |  |  |  |  |
| 24-29 | 66 | 55 (83.3) |  |  |  |  |
| > 29 | 34 | 26 (76.5) |  |  |  |  |
| Living status |  |  | 0.533 |  |  |  |
| Home | 154 | 120 (77.9) |  |  |  |  |
| Assisted living accommodation | 3 | 2 (66.7) |  |  |  |  |
| Hospital admissions <6 months |  |  | 0.643 |  |  |  |
| No | 79 | 61 (77.2) |  |  |  |  |
| Yes | 76 | 61 (80.3) |  |  |  |  |
| Preoperative need for support with activities of daily living |  |  | 0.776 |  |  |  |
| No | 84 | 67 (79.8) |  |  |  |  |
| Yes, outside home | 22 | 17 (77.3) |  |  |  |  |
| Yes, with housework | 33 | 23 (69.7) |  |  |  |  |
| Yes, with basic activities | 18 | 15 (83.3) |  |  |  |  |
| Preoperative mobility |  |  | 0.030 |  |  |  |
| Independently | 95 | 71 (75.5 |  |  |  |  |
| Independently with walking aid | 57 | 49 (86.0) |  |  |  |  |
| Dependent | 5 | 2 (40.0) |  |  |  |  |
| Preoperative mobility outdoors |  |  | 1.000 |  |  |  |
| Yes | 115 | 89 (77.4) |  |  |  |  |
| Assisted | 38 | 30 (78.9) |  |  |  |  |
| No | 4 | 3 (75.0) |  |  |  |  |
| Use of walking aid |  |  | 0.430 |  |  |  |
| No | 81 | 65 (80.2) |  |  |  |  |
| Yes | 76 | 57 (75.0) |  |  |  |  |
| Diagnosis of |  |  |  |  |  |  |
| Congestive heart disease |  |  | 0.797 |  |  |  |
| No | 120 | 96 (80.0) |  |  |  |  |
| Yes | 28 | 23 (82.1) |  |  |  |  |
| Coronary disease |  |  | 0.571 |  |  |  |
| No | 118 | 90 (76.3) |  |  |  |  |
| Yes | 32 | 27 (84.4) |  |  |  |  |
| Hypertension |  |  | 0.638 |  |  |  |
| No | 43 | 32 (74.4) |  |  |  |  |
| Yes | 109 | 85 (78.0) |  |  |  |  |
| Chronic obstructive pulmonary disease (COPD) |  |  | 1.000 |  |  |  |
| No | 149 | 117 (78.5) |  |  |  |  |
| Yes | 5 | 4 (80.0) |  |  |  |  |
| Asthma |  |  | 0.657 |  |  |  |
| No | 144 | 113 (78.5) | 0.657 |  |  |  |
| Yes | 11 | 8 (72.7) |  |  |  |  |
| Diabetes |  |  | 0.229 |  |  |  |
| No | 106 | 80 (75.5) |  |  |  |  |
| Yes | 50 | 42 (84.0) |  |  |  |  |
| Cerebral stroke |  |  | 0.295 |  |  |  |
| No | 131 | 104 (79.4) |  |  |  |  |
| Yes | 23 | 16 (69.6) |  |  |  |  |
| Preoperative cognitive impairment |  |  | 0.137 |  |  |  |
| No | 108 | 88 (81.5) |  |  |  |  |
| Yes | 48 | 34 (70.8) |  |  |  |  |
| Number of medications |  |  | 0.248 |  |  |  |
| No | 63 | 46 (73.0) |  |  |  |  |
| Yes | 94 | 76 (80.9) |  |  |  |  |
| G8 score |  |  | 0.055 |  |  |  |
| < 12 | 71 | 49 (69.0) |  |  |  |  |
| 12-14 | 76 | 65 (85.5) |  |  |  |  |
| > 14 | 10 | 8 (80.0) |  |  |  |  |
| Clinical Frailty Scale (CFS) |  |  | 0.645 |  |  |  |
| 1-2 | 37 | 29 (78.4) |  |  |  |  |
| 3-4 | 80 | 64 (80.0) |  |  |  |  |
| 5-9 | 40 | 29 (72.5) |  |  |  |  |
| Charlson Comorbidity Index (CCI) |  |  | 0.859 |  |  |  |
| 4-6 | 94 | 74 (78.7) |  |  |  |  |
| > 6 | 63 | 48 (76.2) |  |  |  |  |
| ASA score |  |  | 0.927 |  |  |  |
| 2 | 41 | 32 (78.0) |  |  |  |  |
| 3 | 103 | 80 (77.7) |  |  |  |  |
| 4 | 11 | 8 (72.7) |  |  |  |  |
| Mini-Nutritional Assessment-Short Form |  |  | 0.067 |  |  |  |
| 0-7 | 35 | 24 (68.6) |  |  |  |  |
| 8-11 | 109 | 85 (78.0) |  |  |  |  |
| ≥ 12 | 13 | 13 (100.0) |  |  |  |  |
| Haemoglobin (g/L) |  |  | 0.013 |  |  |  |
| ≤ 120 | 101 | 72 (71.3) |  |  |  |  |
| > 120 | 56 | 50 (89.3) |  |  |  |  |
| Albumin (g/L) |  |  | 0.393 |  |  |  |
| ≤ 30 | 28 | 19 (67.9) |  |  |  |  |
| 31-34 | 47 | 38 (80.9) |  |  |  |  |
| ≥ 35 | 71 | 56 (78.9) |  |  |  |  |
| Estimated glomerular filtration rate (GFR) (ml/min) |  |  | 0.927 |  |  |  |
| < 45 | 36 | 28 (77.8) |  |  |  |  |
| 45-60 | 35 | 28 (80.0) |  |  |  |  |
| > 60 | 86 | 66 (76.7) |  |  |  |  |
| Type of operation |  |  | 0.010 |  |  |  |
| Laparoscopy | 106 | 87 (82.1) |  |  |  |  |
| Open | 51 | 35 (68.6) |  |  |  |  |
| Conversion | 13 | 12 (92.3) |  |  |  |  |
| Surgical complications |  |  | 0.046 |  |  |  |
| No | 122 | 99 (81.8) |  |  |  |  |
| Yes | 35 | 23 (65.7) |  |  |  |  |
| Non-surgical complications |  |  | 0.002 |  |  |  |
| No | 119 | 99 (83.2) |  |  |  |  |
| Yes | 36 | 21 (58.3) |  |  |  |  |
| Severe complication (CD III-IV) |  |  | 0.127 |  |  |  |
| No | 139 | 111 (79.9) |  |  |  |  |
| Yes | 18 | 11 (61.1) |  |  |  |  |
| Discharge from operating hospital |  |  | < 0.001 |  |  |  |
| Home | 86 | 76 (88.4) |  |  |  |  |
| Other medical facilities | 70 | 46 (65.7) |  |  |  |  |
| Readmission |  |  | 0.614 |  |  |  |
| No | 136 | 107 (78.7) |  |  |  |  |
| Yes | 13 | 11 (84.6) |  |  |  |  |

*Death during hospital stay (n=1)

| **Table S5** Factors which were evaluated for postoperative mobility with fully mobile patients one-year after surgery. Missing data (n=6) | | | |  |  |  |
| --- | --- | --- | --- | --- | --- | --- |
|  | **All patients n=95** | **Same or better n=71** | **p-value** | |  |  |
|  |  | **n (%)** |  | |  |  |
| Age, years |  |  | 0.501 | |  |  |
| 80-84 | 55 | 40 (72.7) |  | |  |  |
| 85-89 | 30 | 37 (73.7) |  | |  |  |
| 90- | 10 | 17 (90.0) |  | |  |  |
| Gender |  |  | 0.263 | |  |  |
| Female | 50 | 35 (70.0) |  | |  |  |
| Male | 45 | 36 (80.0) |  | |  |  |
| BMI, kg/m^2^ |  |  | 0.918 | |  |  |
| < 24 | 37 | 27 (73.0) |  | |  |  |
| 24-29 | 39 | 30 (76.9) |  | |  |  |
| > 29 | 19 | 14 (73.7) |  | |  |  |
| Hospital admissions <6 months |  |  | 0.996 | |  |  |
| No | 55 | 42 (76.4) |  | |  |  |
| Yes | 38 | 29 (76.3) |  | |  |  |
| Diagnosis of |  |  |  | |  |  |
| Congestive heart disease |  |  | 0.676 | |  |  |
| No | 80 | 61 (76.3) |  | |  |  |
| Yes | 8 | 7 (87.5) |  | |  |  |
| Coronary disease |  |  | 0.751 | |  |  |
| No | 76 | 55 (72.4) |  | |  |  |
| Yes | 15 | 12 (80.0) |  | |  |  |
| Hypertension |  |  | 0.552 | |  |  |
| No | 28 | 22 (78.6) |  | |  |  |
| Yes | 66 | 48 (72.7) |  | |  |  |
| Chronic obstructive pulmonary disease (COPD) |  |  | 0.239 | |  |  |
| No | 91 | 70 (76.9) |  | |  |  |
| Yes | 1 | 0 (0) |  | |  |  |
| Asthma |  |  | 0.599 | |  |  |
| No | 89 | 67 (75.3) |  | |  |  |
| Yes | 5 | 3 (60.0) |  | |  |  |
| Diabetes |  |  | 0.331 | |  |  |
| No | 66 | 48 (72.7) |  | |  |  |
| Yes | 28 | 23 (82.1) |  | |  |  |
| Cerebral stroke |  |  | 1.000 | |  |  |
| No | 83 | 62 (74.7) |  | |  |  |
| Yes | 10 | 8 (80.0) |  | |  |  |
| Preoperative cognitive impairment |  |  | 0.150 | |  |  |
| No | 70 | 55 (78.6) |  | |  |  |
| Yes | 25 | 16 (64.0) |  | |  |  |
| Number of medications |  |  | 0.315 | |  |  |
| No | 47 | 33 (70.2) |  | |  |  |
| Yes | 48 | 38 (79.2) |  | |  |  |
| G8 score |  |  | 0.049 | |  |  |
| < 12 | 36 | 22 (61.1) |  | |  |  |
| 12-14 | 51 | 43 (84.3) |  | |  |  |
| > 14 | 8 | 6 (75.0) |  | |  |  |
| Clinical Frailty Scale (CFS) |  |  | 0.628 | |  |  |
| 1-2 | 35 | 27 (77.1) |  | |  |  |
| 3-4 | 58 | 43 (74.1) |  | |  |  |
| 5-9 | 2 | 1 (50.0) |  | |  |  |
| Charlson Comorbidity Index (CCI) |  |  | 0.831 | |  |  |
| 4-6 | 65 | 49 (75.4) |  | |  |  |
| > 6 | 30 | 22 (73.3) |  | |  |  |
| ASA score |  |  | 0.793 | |  |  |
| 2 | 31 | 24 (77.4) |  | |  |  |
| 3 | 58 | 43 (74.1) |  | |  |  |
| 4 | 5 | 3 (72.7) |  | |  |  |
| Mini-Nutritional Assessment-Short Form |  |  | 0.025 | |  |  |
| 0-7 | 16 | 9 (56.3) |  | |  |  |
| 8-11 | 67 | 50 (74.6) |  | |  |  |
| ≥ 12 | 12 | 12 (100) |  | |  |  |
| Haemoglobin (g/L) |  |  | 0.003 | |  |  |
| ≤ 120 | 59 | 38 (64.4) |  | |  |  |
| > 120 | 36 | 33 (91.7) |  | |  |  |
| Albumin (g/L) |  |  | 0.337 | |  |  |
| ≤ 30 | 14 | 9 (64.3) |  | |  |  |
| 31-34 | 22 | 15 (68.2) |  | |  |  |
| ≥ 35 | 51 | 41 (80.4) |  | |  |  |
| Estimated glomerular filtration rate (GFR) (ml/min) |  |  | 0.959 | |  |  |
| < 45 | 21 | 16 (76.2) |  | |  |  |
| 45-60 | 21 | 16 (76.2) |  | |  |  |
| > 60 | 53 | 39 (76.7) |  | |  |  |
| Type of operation |  |  | 0.004 | |  |  |
| Laparoscopy / conversion | 73 | 60 (82.2) |  | |  |  |
| Open | 22 | 11 (50.0) |  | |  |  |
| Surgical complications |  |  | 0.069 | |  |  |
| No | 74 | 59 (79.7) |  | |  |  |
| Yes | 20 | 12 (60.0) |  | |  |  |
| Non-surgical complications |  |  | 0.096 | |  |  |
| No | 78 | 61 (78.2) |  | |  |  |
| Yes | 17 | 10 (58.8) |  | |  |  |
| Severe complication (CD III-IV) |  |  | 0.558 | |  |  |
| No | 86 | 65 (75.6) |  | |  |  |
| Yes | 9 | 6 (66.7) |  | |  |  |
| Discharge from hospital |  |  | 0.012 | |  |  |
| Home | 60 | 50 (83.3) |  | |  |  |
| Other medical facilities | 35 | 21 (60.0) |  | |  |  |
| Readmission |  |  | 0.729 | |  |  |
| No | 80 | 60 (75.0) |  | |  |  |
| Yes | 10 | 8 (80.0) |  | |  |  |
